# Column study using modified banana pseudo stem as adsorbent for removal of Pb (II)

**DOI:** 10.1016/j.heliyon.2023.e15469

**Published:** 2023-04-15

**Authors:** Suman Pawar, Shridhar Bagali, Uma K, B.S. Gowrishankar

**Affiliations:** a Department of Chemical Engineering, Siddaganga Institute of Technology, Tumakuru, Karnataka, India; b Department of Petrochem Engineering, Khaja Bandanawaz University, Kalaburagi, Karnataka, India; c Department of Chemistry, Siddaganga Institute of Technology, Tumakuru, Karnataka, India; d Department of Biotechnology, Siddaganga Institute of Technology, Tumakuru, Karnataka, India

**Keywords:** Lead, Column study, Heavy metal, Modified banana pseudo stem powder

## Abstract

Eco-friendly adsorbents such as banana pseudo stem play a fundamental role in the removal of heavy metal elements from the wastewater. Key water resources and chemical industries have been encountering difficulties in removing heavy metal elements using existing conventional methods. The lead-removal process is currently a challenging task for environmental scientists and engineers in terms of cost, effluent disposal, and safety concerns. Hence, this work demonstrates the adsorption of Pb (II) onto modified banana pseudo stem (MBPS) powder as a potential adsorbent to treat different effluents. A characterization of modified banana pseudo-stem powder was performed using scanning electron microscopy (SEM) and Fourier-transform infrared (FTIR) spectroscopy which confirms the material. Experiments carried out using a column process for the removal of lead (II) from an aqueous solution at a fixed concentration of 50 ppm, pH 6 and contact time 120 min. The BET surface area of MBPS was found to be 7.27 m^2^/g. The results showed that the column studies explain better performance for the removal of Pb (II) and the maximum removal was found to be 49% at lower flow rate (5 mL/min) of fixed initial concentration of 50 ppm.

## Highlights

• Eco-friendly adsorbent prepared from banana pseudo stem.
• Successfully Pb (II) adsorption on banana pseudo stem adsorbent.
• Different parameters like pH, initial concentration, adsorbent dose and contact time were studied for optimum value of Pb (II) removal.
• Column studies explain in the removal of Pb (II) up to 49% at flow rate (5 mL/min) of fixed initial concentration 50 ppm.

## Graphical abstract

**Figure:**
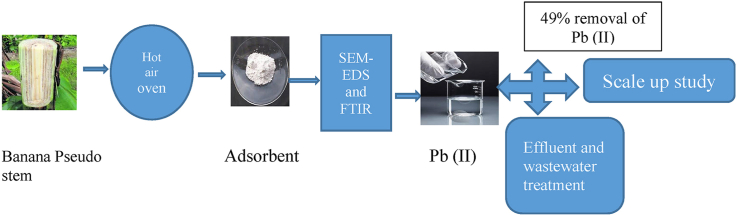

## 1 Introduction

The presence of heavy metals in aqueous waste streams has become a problem due to its harmful effects on human health and to the fauna and flora of receiving water bodies. It is understood that legal, environmental control requirements are becoming stringent and as a result the dumping of heavy metals into marine bodies and drinking water supplies is being rigorously regulated. Wastewater from dyes, pigments, washing of metals, plates [1,2] and effluents from the industries are released into the environment, affecting the purity of water and lead to various syndromes due to the consequential ecological difficulties.

The major sources of Pb (II) in aquatic ecosystem are anthropogenic, including municipal wastewater and industrial effluents discharged from different industries manufacturing batteries, pigments, cables, pipes, ceramics, gasoline, tobacco, steel, food packaging glasses and pesticides [3,4]. Majority of the pollution emitted by the lead produced by the electroplating and pharmaceutical industries to the environment is extremely poisonous in nature and has an awfully harmful effect on biodiversity.

Several conservative techniques such as, electroplating, precipitation, evaporation, membrane separation, ion exchange, coagulation, floatation, reverse osmosis, solvent extraction, membrane filtration, and adsorption, as well as different biological processes, are utilized for the recovery of metals from the effluents [[5], [6], [7], [8], [9]]. Most of these methods are highly laborious and energy consuming; in addition, these techniques have some limitations such as high carbon footprint, high cost, low efficiency, demanding special reagents/chemicals, and challenges associated with the discharge of sludge [9,10]. Hence, research focus should be aimed at continuous processes and at scaling up and meeting the requirements for secondary or tertiary sludge treatment. A wide range of adsorbents have been used to remove heavy metal ions, including Pb(II), such as silica gel [11], activated alumina [12], activated carbon [13,14], fly ash [15], sugarcane bagasse [16], natural clay [17], zeolites [18], nanomaterial's [19,20], polymer based [21] adsorbents fish scales [[22], [23], [24], [25], [26]] and mollusk shells [27,28].

The removal process of Pb is currently a tricky work for researchers in terms of cost, disposal of heavy metal solution and safety concerns [29]. Various predictable water treatment methods employing biosorbents and activated carbon are discussed in many research papers [5,6]. These factors strongly promote the development of cost-effective alternative technologies [4,30,31]. Different adsorption methods are already available but most of the work done on only in batch mode and no reports in the literature about continuous operation. The existing challenge is to use available technology as a real time application by collaborating with chemical related industry for scale up study and implementation of the technology.

The aim of this work is to preparing MBPS from banana pseudo stem, characterizing the MBPS, exploring the influence of adsorption parameters on the rejection of Pb (II) ions from synthetic water, identify the optimal conditions for the removal of Pb (II) for a fixed bed height and at the initial concentration of Pb (II) using MBPS as an adsorbent by column study and study of different models for better fit. Experimentally investigates desorption of heavy metals ions as well as the reusability of the MBPS. Column studies are essential as it provides excellent outcome, exceptionally easy to work, more affordable and can be effortlessly increased from a lab scale to industrial scale. In this work, the adsorbent, MBPS is used in column studies for the treatment of Pb (II). The effluent concentration is one of the foremost characteristics of the fixed bed adsorption. The breakthrough curves are obtained by plotting concentration versus time. The efficiency of the column is explained by the breakthrough curve (BTC) [[32], [33], [34]]. It is reported that, the best experimental data fit for Thomas model compared to the other models and tested the regeneration and reusability of MBPS, and their ability to remove Pb (II) from aqueous solution.

## 2 Materials and methods

All the chemicals were purchased from Sigma-Aldrich and Merck and used without purification.

### 2.1 Adsorbent and reagents

Banana pseudo stem (BPS) was collected from the Gulbarga Banana yard and used for the preparation of MBPS material. The collected samples were ground into powder in domestic blender. Further, powder of BPS was washed several times with distilled water until washed solution turns colourless. The material was dried in hot air oven at 105 °C for 24 h and 80 °C for 8 h respectively. The dried BPS was sieved to obtain the particle size of approximately 500 μm and was stored in an airtight container for further experiments. This untreated biomass directly employed as an adsorbent for the removal of lead. Modification was carried out by collecting 1 g of dried banana pseudo stem powder, which is diluted in 20 mL of 0.1 M of NaOH. This mixture was agitated for 1 h, then washed with distilled water and air-dried. The dried sample was treated with 1.2 M solution of citric acid and at a residue ratio of 8.3 mL solution/gram. For half an hour, mixture was agitated, collected the pellet, washed, and dried at 50 °C for 24 h [35]. The dried sample was stored in an airtight bottle for further experiments.

### 2.2 Stock solution preparation

Solutions with lead ion concentration of 50 ppm were prepared using analytical-grade Pb(NO_3_)_2_. HCl and NaOH buffer solutions of 0.1 N were prepared to adjust the pH of the solutions.

### 2.3 Column adsorption experiments

**Fig. 1:**
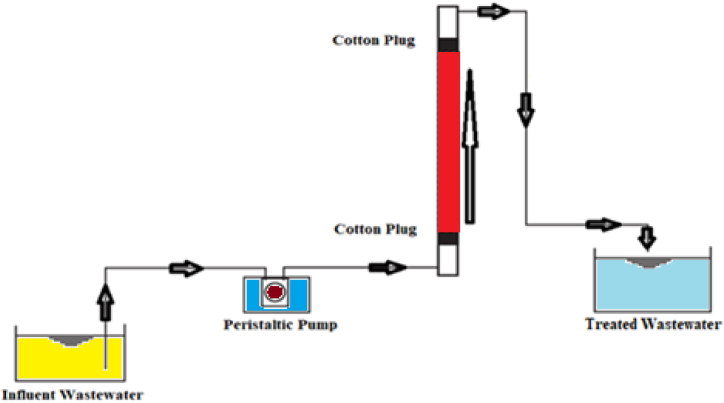
Schematic diagram of column experiments for lead (II) using MBPS.

Column operations were used for the continuous elimination of pollutants from wastewater as this method has several advantages over the batch operation. In the present study, the column experiments were carried out using an acrylic column with a height of 25 cm, 2.5 cm inner diameter, initial concentration of 50 ppm at pH 6 with a contact time of 120 min. The modified pseudo banana stem powder was packed in the column, and the Pb (II) solution was propelled using peristaltic pump (Model: RH-P120VS-2H) in up-flow direction to the column containing MBPS at constant inflow rate. The representation of the column experiments diagram is shown in Fig. 1.

The trials were carried out for different flow rates (5, 7 and 10 mL/min), adsorbent height (6 cm) and at initial Pb (II) solution (50 mg/L). The outflow was collected from the top of the adsorbent filled column at regular intervals, and the residual Pb (II) concentration was measured using Atomic Absorption Spectroscopy (AAS). The process was turned off when the outflow concentration exceeded a value of 99.5% of its initial concentration.

The graph was plotted considering the ratio of C/C_0_ (C and C_0_ are the Pb (II) concentration of effluent and influent, respectively) v/s time used to determine break through curve. The total quantity of Pb (II) absorbed in the bed material (F_ad_) was calculated by multiplying the area above the BTC and the flow rate. The equilibrium uptake capacity (qe, mg/g) of the adsorbent bed was obtained by dividing F_ad_ by the adsorbent mass (M) [36]. The percentage Pb (II) adsorption is obtained using equation (1).

(1) % Removal = (F_ad_/C_0_Qt_e_) * 100

### 2.4 Adsorption mechanism

The heavy metal adsorption from wastewaters by adsorbent material involves few mechanisms such as electrostatic interactions, cation exchange, metal precipitation, and metal reduction followed by sorption, and metal complexation [37]. This process of separation, implies the transfer of adsorbate (i.e., pollutant) from the fluid (i.e., water or industrial effluent) to the surface of a solid matrix (i.e., adsorbent) that have a tailored surface chemistry and porosity to reach an effective separation. It also offers the possibility to recover the adsorbate (s) loaded on the adsorbent surface *via* desorption, thus aiding the adsorbent recycling [38]. The effectiveness of adsorption of heavy metal ions is affected by several operating variables like contact time, adsorbent amount, temperature, initial metal concentration, and pH [39]. Also, textural factors and surface functionalities of the material used as an adsorbent are vital to attain effective removal of the pollutants [40]. Therefore, it is significant to characterize, evaluate, and model the performance of low-cost materials as adsorbents for the removal of heavy metal ions at different operating conditions with the aim of identifying the best alternatives for real-life and industrial applications [41].

## 3 Results and discussion

### 3.1 Characterization of adsorbent

#### 3.1.1 SEM-EDAX analysis of modified banana pseudo stem

**Fig. 2:**
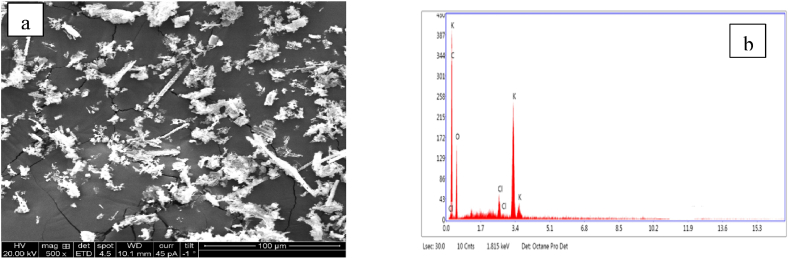
(a): SEM and 2 (b): EDAX images of modified banana pseudo stem powder.

The SEM images (Fig. 2) of modified banana pseudo stem shows the rupture of the surface and the high porosity on the surface helps to enhance the adsorption of the adsorbate. The EDX images of modified BPS suggest that C and O contributed to the two main elements of biomass.

#### 3.1.2 FTIR study of modified banana pseudo stem

**Fig. 3:**
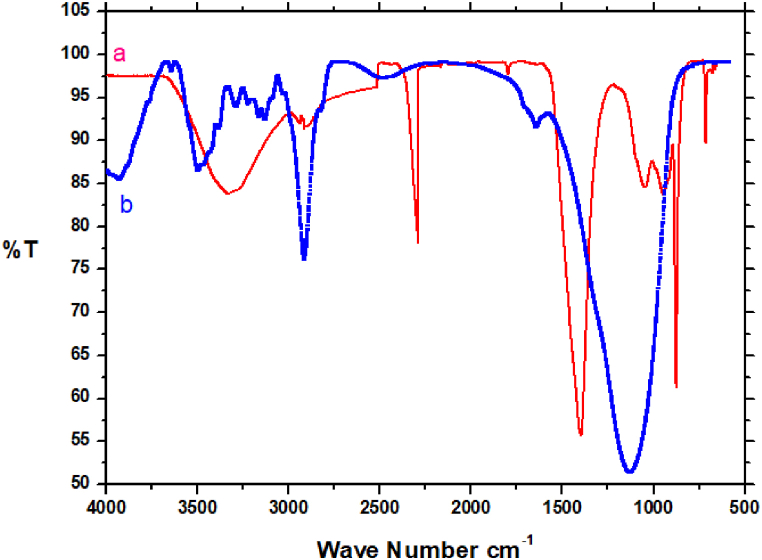
FTIR spectrum of modified BPS (a) before (b) after adsorption.

FTIR spectra before and after Pb^2+^ adsorption confirms the nature of Pb^2+^ adsorption (Fig. 3). A small N–H vibration band shift from 3250 to 3300 cm^−1^ is observed after Pb^2+^ adsorption. This indicates the attachment of Pb^2+^ to the N group affecting the N–H vibration. Peaks in the spectrum at 3600, 1760, 1360 and 1100 cm^−1^ region are related to the O–H stretching, C=O, –CH_3_, and C–N stretching bands respectively [42].

### 3.2 Breakthrough curve studies

**Fig. 4:**
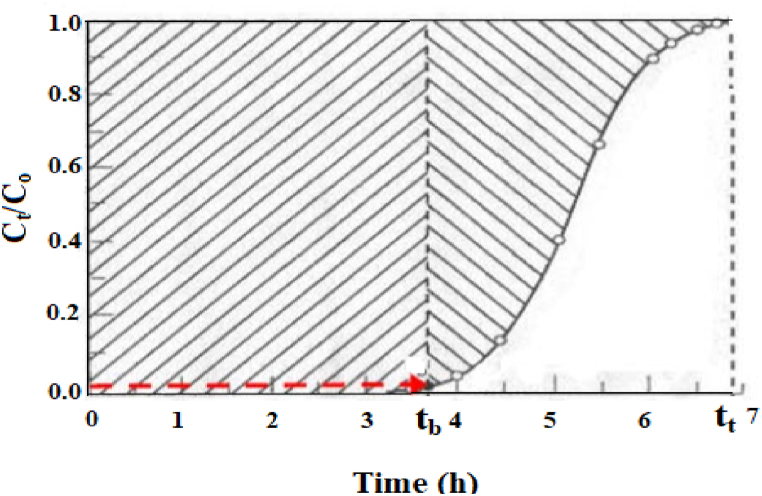
Breakthrough curve as a function of contact time.

The specific profile of the BTC depends on few factors comprising of the inlet flow rate, initial solute concentration (IC) and bed height (BH) [43,44]. The adsorption ability of a packed column of fixed proportions depends on the equilibrium between the solute and adsorbent, upon the mechanism of transfer and the adsorption rate [45]. In the present study, the shape of the curve shows that adsorption is mainly restricted by mass transfer depicted in Fig. 4.

#### 3.2.1 The effect of initial flow rate

**Fig. 5:**
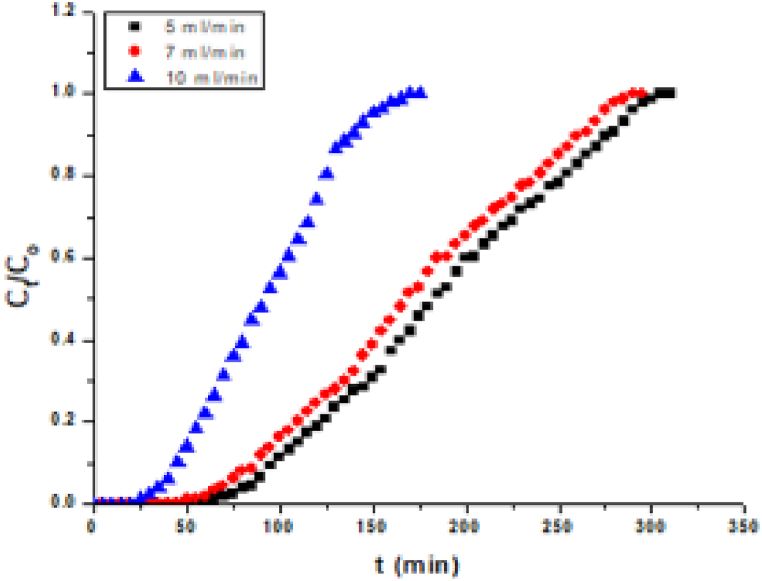
Comparison of predicted breakthrough curves for MBPS adsorption of Pb (II).

The effect of influent flow rate on the adsorption of Pb (II) on MBPS was studied by varying the flow rate (5, 7 and 10 mL^−1^min) while the initial concentration and bed height is kept constant i.e. 50 ppm and 6 cm respectively. The BTC of the comparative metal ion concentration (C_t_/C_o_) versus time (min) at different flow rates is shown in Fig. 5 for the removal of Pb (II) using MBPS. From the experiment, it is found that, the BTC occurred vary fast with greater flow rate on Pb (II) compounds eluted rapidly as the flow rate increased.

The breakthrough time has been decreased from 80 to 35 min with increased flow rate from 5 to 10 mL/min for MBPS on adsorption of Pb (II). This may be due to the increased flow rate of an adsorption zone which resulted in a decrease in the time needed to reach the specific concentration of the breakthrough. It is observed that, increase in the flow rate from 5 to 10 mL/min decreases the percentage removal from 49 to 36% of Pb (II) using MBPS powder. The rate of mass transfer increases with increase in flow rate, which helps in enhancing the rate of adsorption. Because of the higher flow rate, the earlier result of the breakthrough point occurs. Further, the retention time of Pb (II) molecules in the column depends on the flow rates. Because of the higher flow rate, it decreases the contact time from 310 to 175 min for Pb (II) using MBPS (Table 1). Therefore, at a higher flow rate, the removal percentage is lesser and it is difficult to reach the equilibrium. Breakthrough performance is analyzed by utilizing different models. These models help in calculating adsorption capacity and column kinetics of the adsorbent bed. The kinetics of adsorption is studied using various models including the Thomas model (TM) and Yoon-Nelson (YN) model. The results of breakthrough curves were compared with other agricultural waste used for the removal of Pb (II) and were in agreement with the reported results [46,47]. Overall results showed that the column studies explain better performance in the removal of Pb (II) at lower flow rates.

**Table 1:** Parameters of breakthrough curves for Pb (II).

Parameters	Pb (II)		
FR (mL/min)	5	7	10
t_b_ (min)	80	65	35
t_t_ (min)	310	295	175
t_0.5_ (min)	185	170	95
% removal	49	43	36

### 3.3 Thomas model (TM) on MBPS for the treatment of Pb (II)

**Fig. 6:**
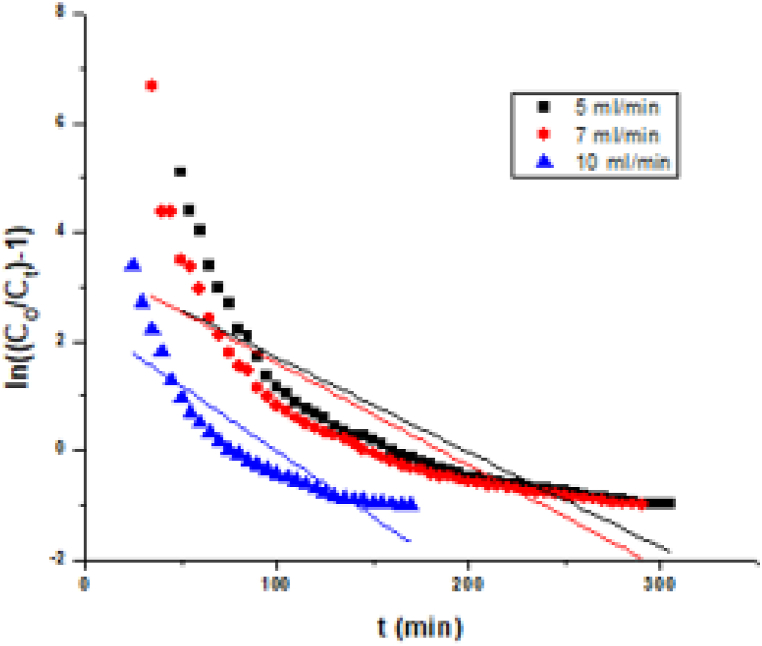
Thomas model parameters for MBPS on adsorption of Pb (II).

Theoretical model proposed by Thomas is widely used to describe the column performance. Thomas model is most simple and extensively used by quite a few investigators [48,49]. The data found from column studies fitted with the model to determine the kinetic coefficient (K_Th_) and adsorption capacity (q_o_) [50]. The K_Th_ and q_o_ can be calculated from Fig. 6 for the removal of Pb (II) using MBPS.

The q_o_ decreases from 29 to 17 (mg/g) and the K_Th_ also called as Thomas rate constant which increases from 0.66 to 0.87 (mL/min mg) with increased flow rate from 5 to 10 (mL/min) as shown in Table 2 for MBPS on adsorption of Pb (II). The coefficient of correlation (R^2^ = 0.96), suggesting that this model resulted in a good fit to the experimental data compared to Yoon Nelson model. The obtained results were compared with other studies where agricultural waste used for the removal of Pb (II) is as shown in (Table 2) [[51], [52], [53], [54], [55]].

**Table 2:** Thomas parameters for MBPS on adsorption of Pb (II).

Thomas Parameters	Pb (II)		
Flow rate (mL/min)	5	7	10
K_Th_ (mL/min mg)	0.66	0.77	0.87
q_o_ (mg/g)	29	21	17
R^2^	0.9659	0.9310	0.9557

### 3.4 Yoon – Nelson model

**Fig. 7:**
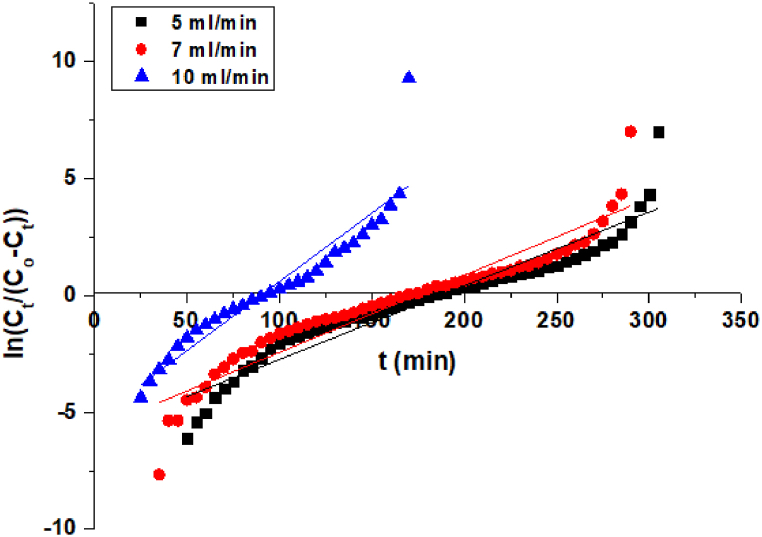
Yoon - Nelson parameters for MBPS on adsorption of Pb (II).

This model has the ability to predict the time for 50% adsorbate breakthrough. The linearized form of the Yoon–Nelson model plot is presented in Fig. 7. Yoon-Nelson model application is a simple theoretical model, used for testing Pb (II) breakthrough actions on the MBPS. The values of Yoon–Nelson constant K_YN_ (min^−1^) and τ (min) were obtained from Fig. 7 for the removal of Pb (II) using MBPS.

According to the outcomes K_YN_ has been increased from 0.026 to 0.035 (min^−1^) as flow rate increases from 5 to 10 (mL/min), whereas the 50% of τ reduced from 202 to 91 min with an increase in flow rate as depicted in Table 3. The lower the K_YN_ value result revealed that the mass transfer by the diffusion inside the column was better and higher the K_YN_ value indicated the lower diffusion for the column [[51], [52], [53]].

**Table 3:** Yoon - Nelson parameters for MBPS on adsorption of Pb (II).

Yoon - Nelson Parameters	Pb (II)		
Flow rate (mL/min)	5	7	10
K_YN_ (min^−1^)	0.026	0.029	0.035
τ(min)	202	174	91
R^2^	0.9131	0.9015	0.9186

## 4 Reusability and regeneration of the material

Regeneration of adsorbent has an important significance in economic and environmental point of view. Regeneration studies can aid in understanding the adsorption process between the adsorbent and adsorbate [[56], [57], [58]].

**Fig. 8:**
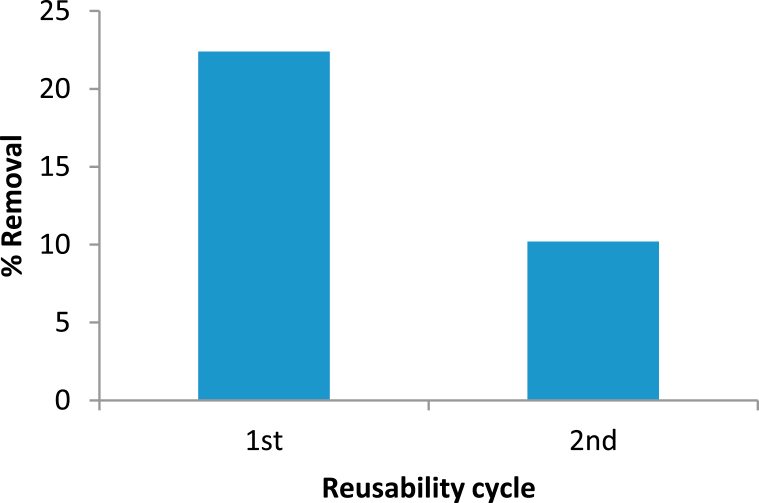
Repeatability of the Pb^2+^ adsorbent using MBPS.

In the present study, 0.5 M HCl was used as a desorbing agent for regeneration because adsorbed lead forms a thin film of on the external surface of the adsorbent that can be easily dissolved in HCl [59]. The adsorbent regenerated by this method were reused for further adsorption/desorption cycles to examine how well they can retain their metal adsorption capacity. The repeatability of the Pb^2+^ adsorbent made from MBPS is shown in Fig. 8.

The results showed a success rate of 22.4% for the first cycle and a success rate of 10.2% for the second cycle. The declining trend might be the consequence of diminishes the surface area and block the available active site/binding area which might be a chemical reaction which is not easily reversible [[60], [61], [62]]. The regeneration efficiency was calculated for two cycles among these very effective removal of Pb (II) found for the first cycle.

## 5 Conclusion

Modified banana pseudo stem adsorbent performed well for the Pb (II) adsorption employing column studies. The physicochemical and structural characteristics of developed adsorbent were determined by the SEM-EDX,FT-IR and BET analysis in order to know crystalline structure, functional groups and elements present in the adsorbent and surface area of the material. The results report that flow rate of the feed to column equal to 5 mL/min, shows 49% removal compared to higher flow rates. Hence, lower the flow rate, removal of Pb percentage increases. So, for the initial concentration of 50 mg/L of Pb (II), high removal is achieved compared to (>50 mg/L) for the bed height of adsorbent 6 cm. It is found that the Thomas model fits well to experimental data for Pb (II) adsorption on porous MBPS. The spent adsorbent can be reused for successive two cycles after regenerating the adsorbent. Hence, the MBPS is an excellent material in treating wastewater and industry effluent samples.

### 5.1 Future work

Material selectivity performance and stability can be carried out for real time applications.

## Author contribution statement

**Suman Pawar:**Conceived and designed the experiments; Wrote the paper.

**Shridhar Bagali**: Performed the experiments.

**Uma K:** Analyzed and interpreted the data; Wrote the paper.

**B. S. Gowrishankar:** Contributed reagents, materials, analysis tools or data.

## Data availability statement

The data that has been used is confidential.
